# Nanofabrication of Topological Surface Relief Structures in Azopolymers With Tightly Focused Optical Skyrmions

**DOI:** 10.1002/nap2.70347

**Published:** 2026-10-30

**Authors:** Takashige Omatsu, Soki Hirayama, Andra Naresh Kumar Reddy, Masahito Okida, Atsushi Taguchi

**Affiliations:** ^1^ Molecular Chirality Research Center Chiba University Chiba Japan; ^2^ Department of Electrophysics National Yang Ming Chiao Tung University Hsinchu Taiwan; ^3^ Institute of Laser and Optoelectronics School of Precision Instrument & Optoelectronics Engineering Tianjin University Tianjin China; ^4^ Serendip Research Osaka Japan; ^5^ Osaka Metropolitan University Osaka Japan; ^6^ Tohoku University Sendai Japan; ^7^ Research Institute for Electronic Science Hokkaido University Sapporo Japan

## Abstract

We demonstrate the nanofabrication of topological surface relief structures (surface reliefs with a shape like a Möbius strip) in azopolymers using longitudinal electric fields and nano‐scale circular polarization defects (C‐point polarization defect) created by tightly focused optical skyrmions. The fabricated surface reliefs exhibited structures with a shape like a Möbius strip, reflecting the topologically protected polarization textures (Möbius strip shaped electric field textures) of optical skyrmions. Also, the surface reliefs fabricated with Bloch skyrmions were twisted, becoming chiral, due to orbital angular momentum (OAM) transfer effects. Furthermore, a two‐dimensional (2D) array of nano‐scale topological surface reliefs was formed via the application of a 9 × 9 spot matrix diffractive optical element (DOE).

## Graphical abstract

We demonstrate the nanofabrication of topological surface relief structures (surface reliefs with a shape like a Möbius strip) in azopolymers using longitudinal electric fields and nano‐scale circular polarization defects (C‐point polarization defect) created by tightly focused optical skyrmions. Also, the surface reliefs fabricated with Bloch skyrmions were twisted, becoming chiral, due to orbital angular momentum (OAM) transfer effects.

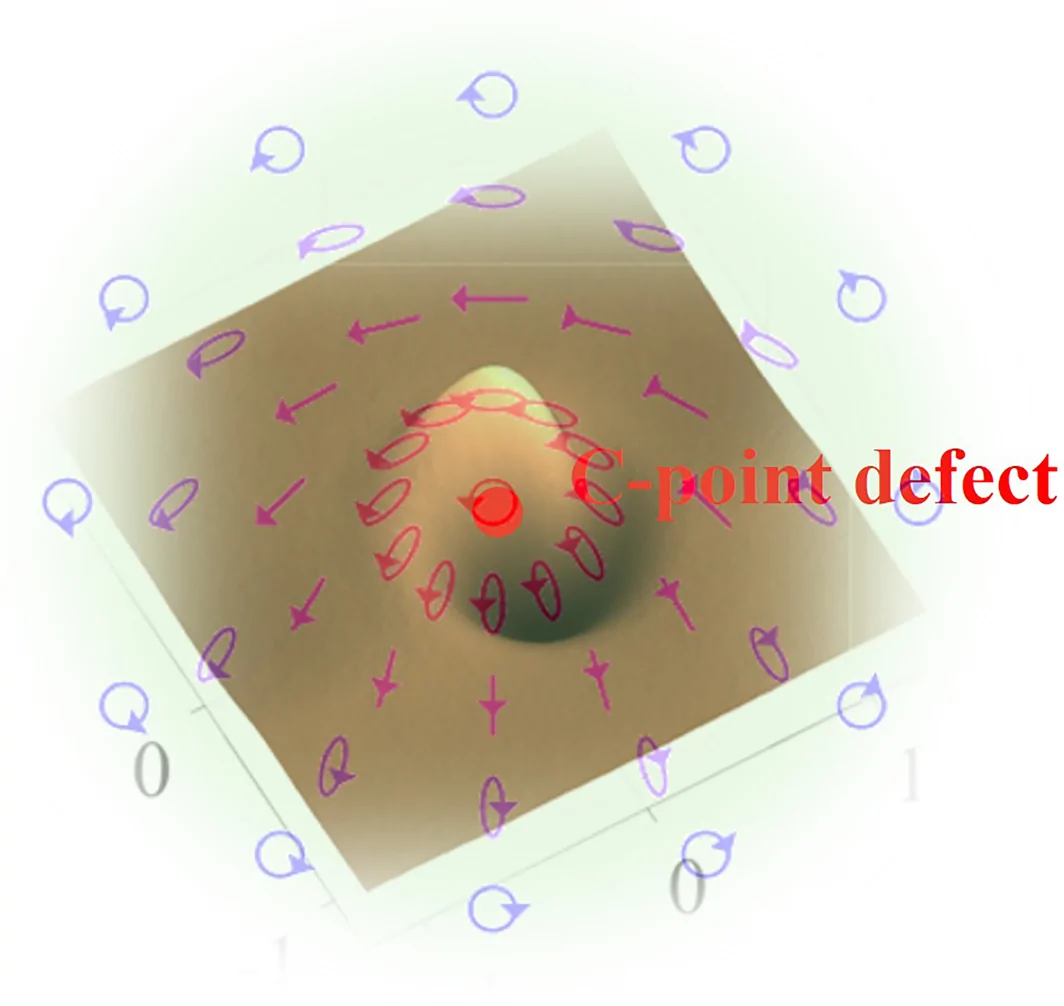

## 1 Introduction

Chirality is a characteristic wherein a material cannot be superimposed onto its mirror image; and it is a universal and important property in materials science [1, 2].

Structured light beams possess orbital angular momentum (OAM) (characterized by a topological charge *l*) [3, 4, 5, 6], which is associated with their helical wavefronts. They offer numerous advantages across diverse applications, such as super‐resolution microscopy [7], optical trapping and manipulation [8, 9, 10], and space‐division multiplexing classical/quantum optical communication [11, 12]. Furthermore, it has been discovered that the OAM of structured light beams twists materials to produce micro‐scale chiral structures, with the help of the spin angular momentum (SAM) (characterized by *σ* = ± 1) of circularly polarized light with a helical electric field [13, 14].

In recent years, two‐dimensional and three‐dimensional (2D or 3D) particle‐like optical waveforms, which have a spatially inhomogeneous wavefront or polarization in physical space and time (topologically protected wavefront or polarization textures), have emerged as a new class of structured light. These are optical quasiparticles, often referred to as optical skyrmions and optical bimerons [15].

Optical skyrmions are remarkable optical quasiparticles and are considered optical counterparts of magnetic spin skyrmions with topologically protected spin textures. Néel, Bloch, and anti‐skyrmions, have enabled new approaches to information encoding and the development of low‐energy magnetic memory devices [16, 17]. Optical skyrmions can be formed through the coherent superposition of fundamental (Gaussian) and Laguerre–Gaussian (LG) modes with orthogonally circular polarizations [18, 19, 20, 21], and they encompass all polarization states (Stokes vectors of paraxial vector beams) represented by a Poincaré sphere. Thus, Néel and Bloch optical skyrmions also exhibit chirality.

To date, several approaches for generating optical skyrmions have been experimentally demonstrated. These include polarization interferometry based on a spatial light modulator [22, 23] or an intracavity wedge‐plate shearing interferometer configuration [24, 25], a custom‐designed *q*‐plate with different engineered topologies along its radial and azimuthal directions [26], and a spin‐orbit conversion control approach with a single *q*‐plate [27].

Optical skyrmions are rapidly finding utility in advanced applications, such as free‐space communications under turbulent conditions (polarization textures are invariant under spatial propagation through turbid media) [28], polarization nanoscale‐metrology [29], and super‐resolution microscopy with a subwavelength spatial resolution. Furthermore, they possess the capacity to impart their unique polarization characteristics into a material: a process which may enable new methods in chiral materials processing and manipulation.

We have experimentally demonstrated the direct imprint of topologically protected polarization textures of paraxial optical skyrmions onto a photosensitive material (azopolymer). Here, the fabricated surface structures have taken on the lemon‐ or star‐shaped polarization textures of the incident optical skyrmions [30, 31].

In contrast to more conventional structured light beams such as optical vortices and vector beams, quasiparticles of light (i.e., optical skyrmions) are still in their infancy as a research topic. Except for our above‐mentioned demonstrations with paraxial optical skyrmions and mapping of skyrmion topology from a laser beam onto a gas of cold atoms [32], there have been very few reports detailing the interaction between optical skyrmions and materials, such as chiral materials manipulation and fabrication based on unique topological polarization structures of optical skyrmions.

In this work, we demonstrate the nano scale (subwavelength scale) fabrication of chiral surface structures with a shape like a Möbius strip in a photosensitive azopolymer film with tightly focused optical skyrmions. Our approach extends beyond the paraxial condition, in which a focused optical skyrmion twists the azopolymer around its C‐point polarization defect to form surface structures that reflect the topological (chiral Möbius strip shaped) polarization (electric field) textures of optical skyrmions via SAM‐OAM coupling effects.

Möbius strip‐shaped structures are an example of an unconventional chiral structure with no defined front or back. Nano scale Möbius strip‐shaped materials should possess exotic electromagnetic properties (for instance, magnetic monopole etc.), and mechanical properties (for instance, wear‐resistance, etc.), and thus, they have long been of interest. In fact, molecular Möbius strips enable the change of electron cloud distributions in frontier molecular orbitals, so as to affect the electron spin interactions between different orbits. Thus, chemists believe that Möbius strip‐shaped nano or submicron dimension materials may be considered the “ultimate” chiral materials [33].

The work herein highlights the fact that optical skyrmions have the potential to manipulate the chirality and topology of materials, a process, which may enable advanced nanofabrication techniques and achieve a high spatial resolution which extends beyond the diffraction limit.

## 2 Experiments

Figure 1 shows the experimental setup which utilizes a single‐path polarization shaping configuration with a single spatial light modulator (SLM) as shown in our previous publication [34]. A collimated continous‐wave (CW) green laser (wavelength: 532 nm, average power: ∼100 mW) was used, and it exhibited a Gaussian spatial form and a linear polarization inclined at approximately 45° relative to the horizontal axis by using a half‐wave plate (HWP). The laser output was directed toward a SLM (X10468‐04, 792 × 600 pixels, pixel size of 20 × 20 μm^2^, Hamamatsu co.) with an azimuthal spiral 2π phase mask. Only its *x*‐polarized component was converted into an LG mode with radial and azimuthal indices *p* = 0 and *l* = ± 1, and its *y*‐polarized component remained a Gaussian beam.

**FIGURE 1:**
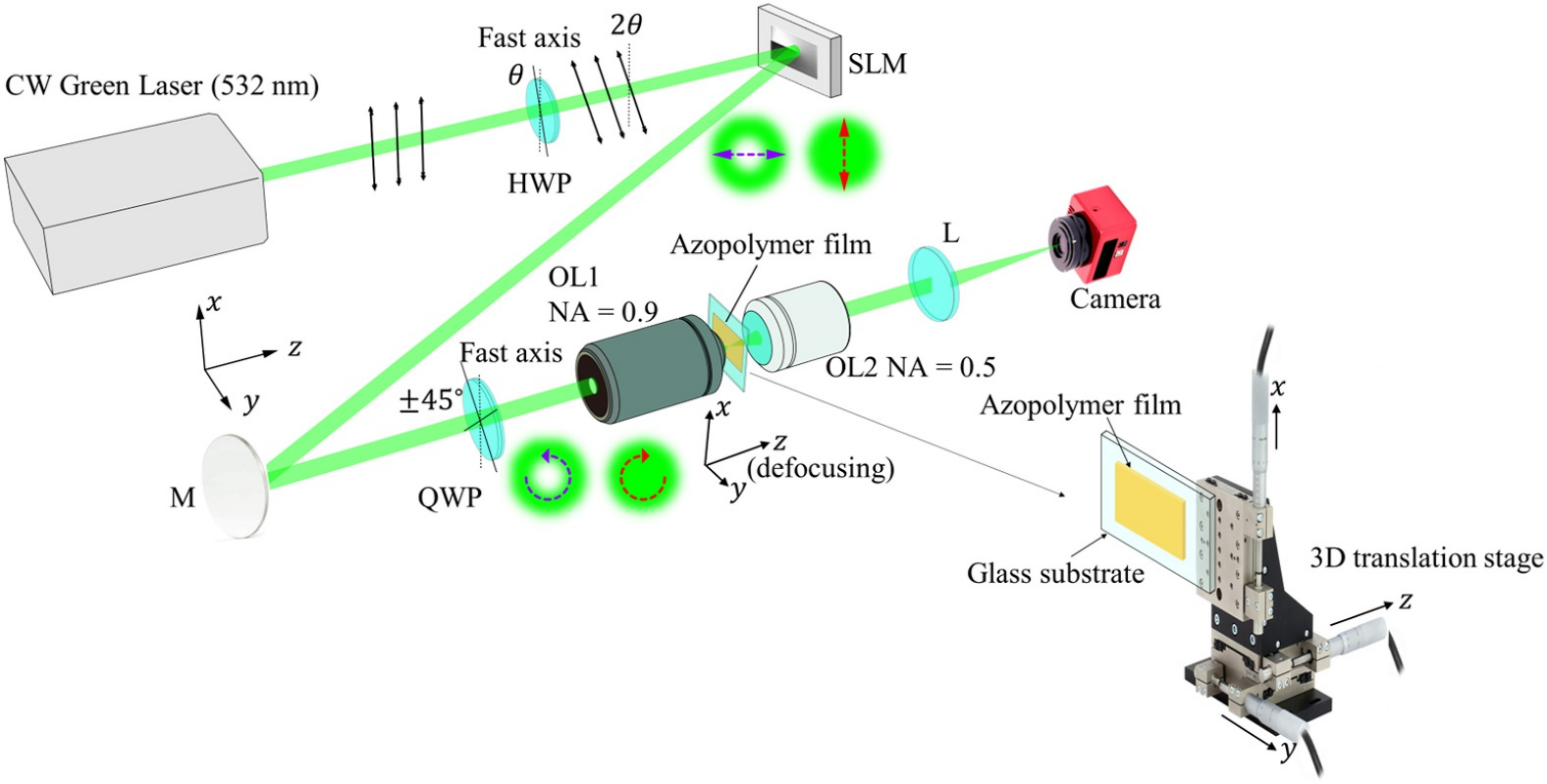
Schematic of the experimental setup used for the fabrication of surface‐relief structures. First order optical skyrmions are generated using a spatial light modulator (SLM) and a quarter‐wave plate (QWP), and they are tightly focused onto an azopolymer film via an objective lens (OL1) with NA = 0.9. The film irradiated with the skyrmion spot is imaged onto a CCD camera by using an objective lens (OL2, NA = 0.5) and a lens (L, *f* = 300 mm). HWP: half‐wave plate, M: total reflection mirror.

The optical field S(r,ϕ) of an optical skyrmion can be described by

(1) $$S\left(r,\phi \right)={\mathrm{LG}}_{0}\left(r,\phi \right)\left|0\rangle \cos\left(\theta \right)e^{-i\beta /2}+{\mathrm{LG}}_{l}\left(r,\phi \right)\right|1\rangle {\sin\left(\theta \right)e}^{i\beta /2},$$

where ${\mathrm{LG}}_{l}\left(r,\phi \right)$ is the optical field of ℓth order LG mode (0th order LG mode with *l* = 0 being a fundamental Gaussian mode), *r* and *ϕ* are the radial and azimuthal coordinates, *θ* and *β* are the mixing parameters and the relative angle between the Gaussian and LG modes, respectively, and |0> and |1> are the orthogonal circular polarizations, respectively.

The skyrmion number *N*_*sk*_ is given by

(2) $$N_{sk}=\frac{1}{4\pi }\iint_{S}s\cdot \;\left(\frac{\partial s}{\partial x}\times \frac{\partial s}{\partial y}\right)dxdy$$

where ***s* **= (*S*_1_, *S*_2_, *S*_3_) is the Stokes vector forming a quasiparticle confined in a sphere *S*.

In our experiments, a quarter‐wave plate (QWP1) enables straightforward generation of optical skyrmions via transformation from the linear polarization basis to the circular polarization basis. Note that |ℓ| is fixed at 1. The power ratio *α* = (1/tan^2^(*θ*/2)) between Gaussian and LG modes was tuned to be 1:1.5 (=0.67) by adjusting the orientation angle of the HWP. *β* was also controlled by the azimuthal orientation of the spiral phase mask on the SLM, and it was fixed at π/2. The spatial intensity profiles, Stokes vector profile, and Stokes parameters of the generated optical skyrmions are shown in Figure 2. Also, it is worth mentioning that Bloch skyrmions (it is equivalent to Néel skyrmions in terms of optical properties, and it exhibits a vortex Stokes vector profile) and anti skyrmions (with saddle Stokes vector profile) are chiral, and their handedness was here defined by the sign of topological charge (OAM) of the LG mode. Experimental left‐handed (wavefront (*l* = 1) and circular polarization (*σ* = −1) of the LG mode had the opposite handedness) Bloch skyrmions exhibited lemon‐shaped polarization textures with a C‐point polarization defect, and their skyrmion number *N*_*sk*_ was measured to be +0.8. Also, experimental anti‐skyrmions (*l* = −1, *σ* = −1) exhibited star‐shaped polarization textures with a C‐point polarization defect, and its skyrmion number *N*_*sk*_ was also measured to be −0.8.

**FIGURE 2:**
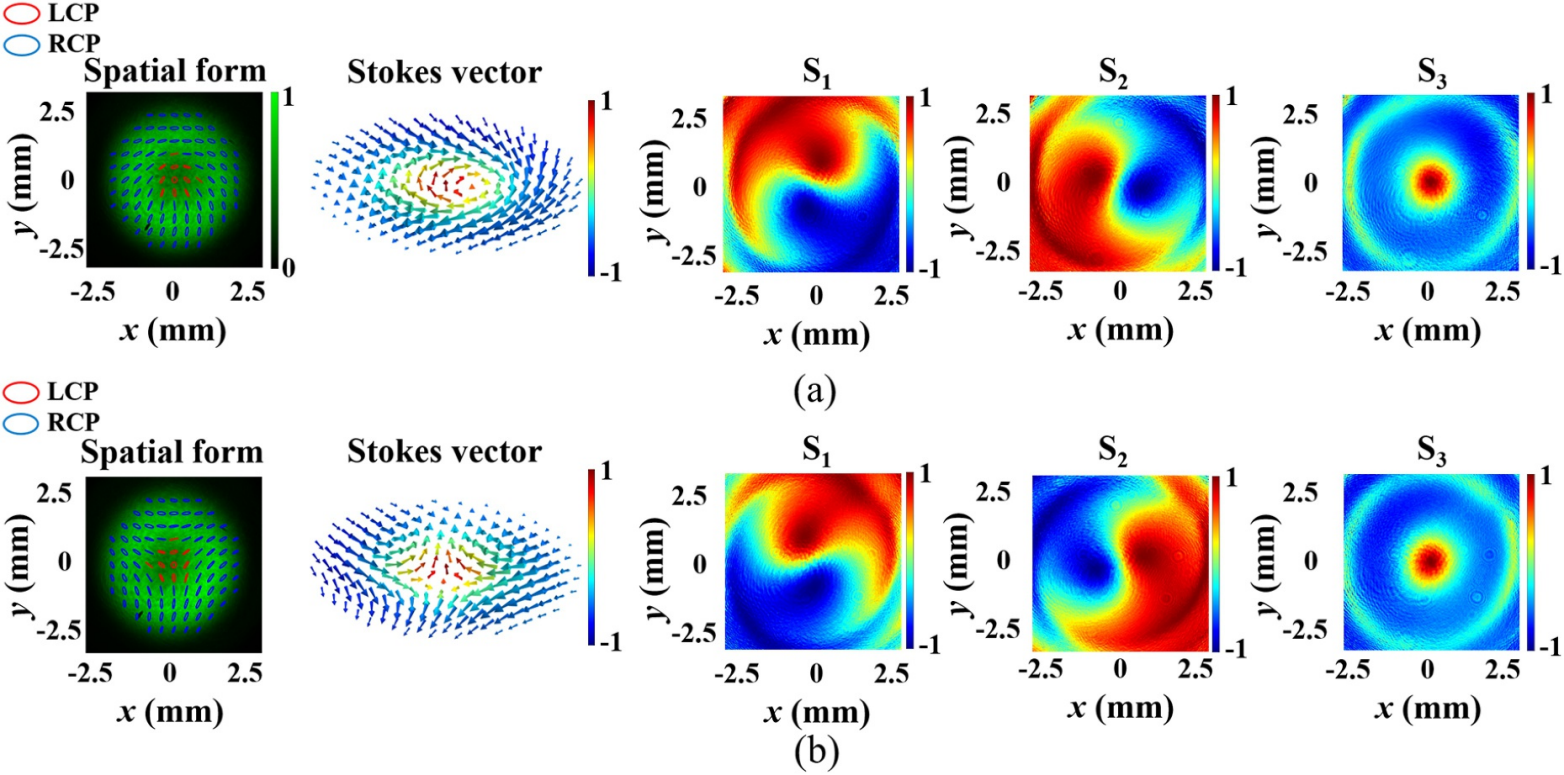
Polarization states of Bloch and anti‐skyrmions in free space. (a) Spatial intensity and polarization profiles, Stokes vector profile, and Stokes parameters (S_1_, S_2_, S_3_) of a Bloch skyrmion. The skyrmion number is estimated to be ∼0.8. (b) Spatial intensity and polarization profiles, Stokes vector profile, and Stokes parameters (S_1_, S_2_, S_3_) of an anti‐skyrmion. The skyrmion number is estimated to be ∼ ‐0.8.

These values were limited by an elliptical (imperfect circular) polarization state at the edge of optical fields. Decreasing the value of *α* will enable the generation of a nearly perfect circular polarization state at the edge of optical fields, and it yields an improvement of the skyrmion number; however, the generated skyrmions then will possess a central intensity dip.

Azopolymers exhibit photoinduced mass transport from the bright region of the irradiating light, toward the dark region, following the polarization direction of the irradiating light field via the photo‐isomerization process, so as to form unique surface relief structures [35, 36, 37, 38]. Surface relief structures fabricated in azopolymers, in general, reflect the spatial intensity and polarization profiles of the irradiating light field.

In this work, optical skyrmions were tightly focused to a spot with a diameter of ∼2 μm onto an annealed azopolymer film (poly‐orange tom‐1 (POT), ∼1 μm thickness) which was spin‐coated onto a UV‐cleaned glass substrate by using an objective lens with NA∼0.9. The laser power and exposure time of the irradiating optical skyrmions were fixed at 0.4 μW and 2.5 seconds, respectively. The fabricated surface relief structures were characterized using an atomic force microscope (AFM; SHIMADZU, scanning probe microscope SPM9700; spatial resolution 30 nm). Note that the azopolymer film used in the experiments possessed moderate absorption (optical density ∼ 2) at 532 nm.

Figure 3 shows AFM images of surface relief structures fabricated with Bloch skyrmions, including 3D skeleton views. Left‐handed Bloch skyrmions enabled the fabrication of a teardrop shaped, clockwise twisted (chiral) surface relief around the C‐point defect, which reflects the lemon‐shaped polarization texture and OAM of the irradiating Bloch skyrmion. Note that the diameter, height and surface‐modulation depth of the fabricated relief were measured to be < 1 μm, ∼100 nm, and ∼170 nm, respectively. Surprisingly, the peak of the relief structure was directly connected with the relief valley around the C‐point polarization defect. This is a manifestation of the π phase jump of polarization textures. Furthermore, when the handedness of the Bloch skyrmion was inverted, the direction of twist of the surface relief was also inverted. These results highlight that the topological (Möbius‐strip‐shaped) polarization (electric field) textures of the irradiating Bloch skyrmions produce exotic chiral surface relief structures resembling a Möbius strip on the azopolymer film, and they have never been observed in our previous work under a paraxial condition.

**FIGURE 3:**
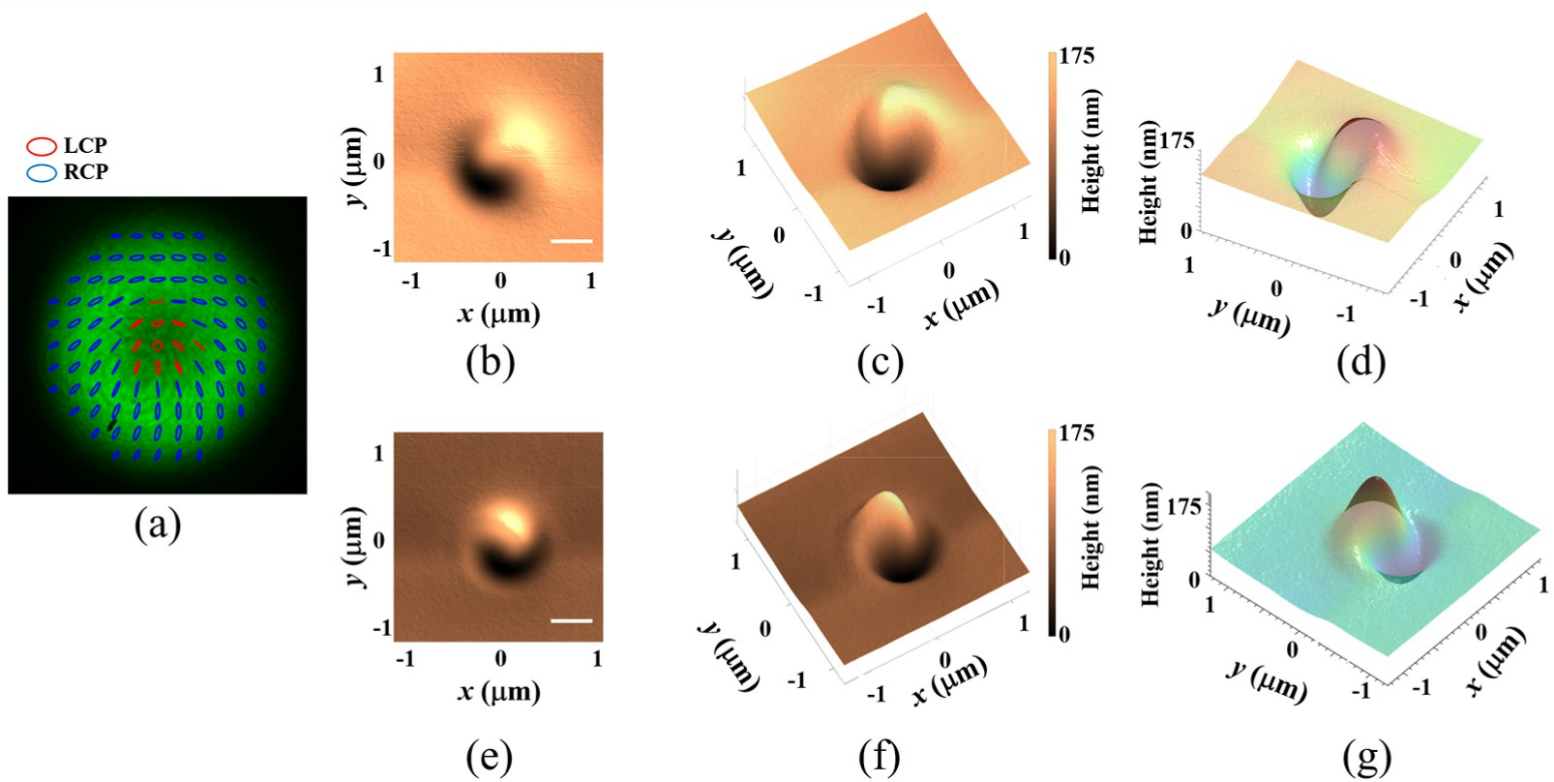
Images of fabricated surface‐relief structures formed using tightly focused (a, b, c) left‐handed Bloch and (d, e, f) right‐handed Bloch skyrmions. (a) Spatial intensity and polarization profile of irradiated Bloch skyrmion. (b, e) 2D AFM images, (c, f) 3D AFM images, and (d, g) 3D skeleton view AFM images. Both surface reliefs are twisted in a counterclockwise or clockwise direction, demonstrating azimuthal mass transport in azopolymers driven by the nano‐scale circular polarization field arising from the spin‐orbit interaction of a tightly focused optical skyrmion. The peak of the relief structure is directly connected with the valley of the structure around the C‐point polarization defect. This is a manifestation of the π phase jump of polarization textures. The solid white line is a 0.5 μm scale bar.

Anti‐skyrmions produced an azimuthally three‐folded surface relief with three petals and a deep central dip with a diameter of ∼300 nm below the diffraction limit. The structures manifested star‐shaped polarization textures around the subwavelength scale C‐point polarization defect, regardless of their sign of *l*. The surface‐modulation depth of the structures was measured to be ∼100 nm (Figure 4). It is worth noting that Bloch and anti skyrmions have identical spatial intensity profiles in the paraxial region; however, they produced completely different surface reliefs, which reflected not intensity profile but polarization textures of irradiated optical skyrmions.

**FIGURE 4:**
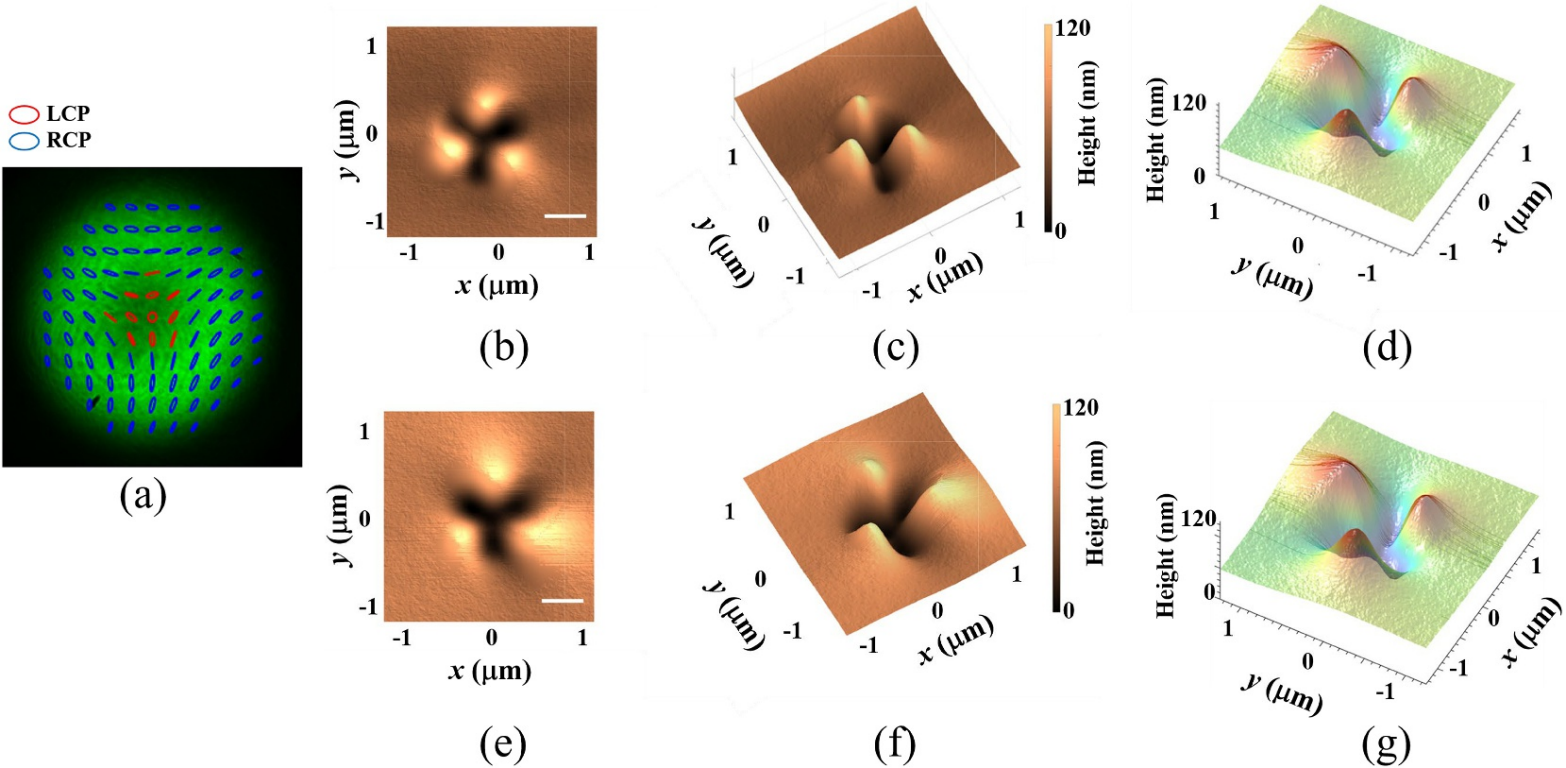
Images of fabricated surface‐relief structures formed using tightly focused anti‐skyrmions with (a, b, c) ℓ = −1 and (d, e, f) ℓ = 1. (a) Spatial intensity and polarization profile of irradiated anti‐skyrmion. (b, e) 2D AFM images, (c, f) 3D AFM images, and (d, g) 3D skeleton view AFM images. Both surface relief structures are achiral (nontwisted) and three‐folded, thus resembling star‐shaped polarization textures of anti‐skyrmions. The reliefs also possess a subwavelength (∼300 nm) central dip around the C‐point polarization defect; this demonstrating an example of subwavelength scale, nanofabrication using this process. The solid white line is a 0.5 μm scale bar.

The topological structure of fabricated surface reliefs remained unchanged for all tested laser powers and exposure times; however, higher laser power and longer exposure time (of the irradiating optical skyrmions) increased the modulation depth of the structures.

When the Bloch and anti‐skyrmion fields (*l* = −1) were defocused on the film by shifting the film on‐axis, the fabricated surface relief structures were rotated in the counterclockwise direction owing to the Gouy phase shift at the defocused region (|*z|* ≤ 1 μm). The surface modulation depth then decreased. Notably, the surface relief structures fabricated using Bloch skyrmions were transformed into nontwisted ones with a shallow dip owing to the disappearance of longitudinal electric field effects at the defocused region. This means that the topological protection of polarization textures is valid only under the paraxial condition (Figure 5).

**FIGURE 5:**
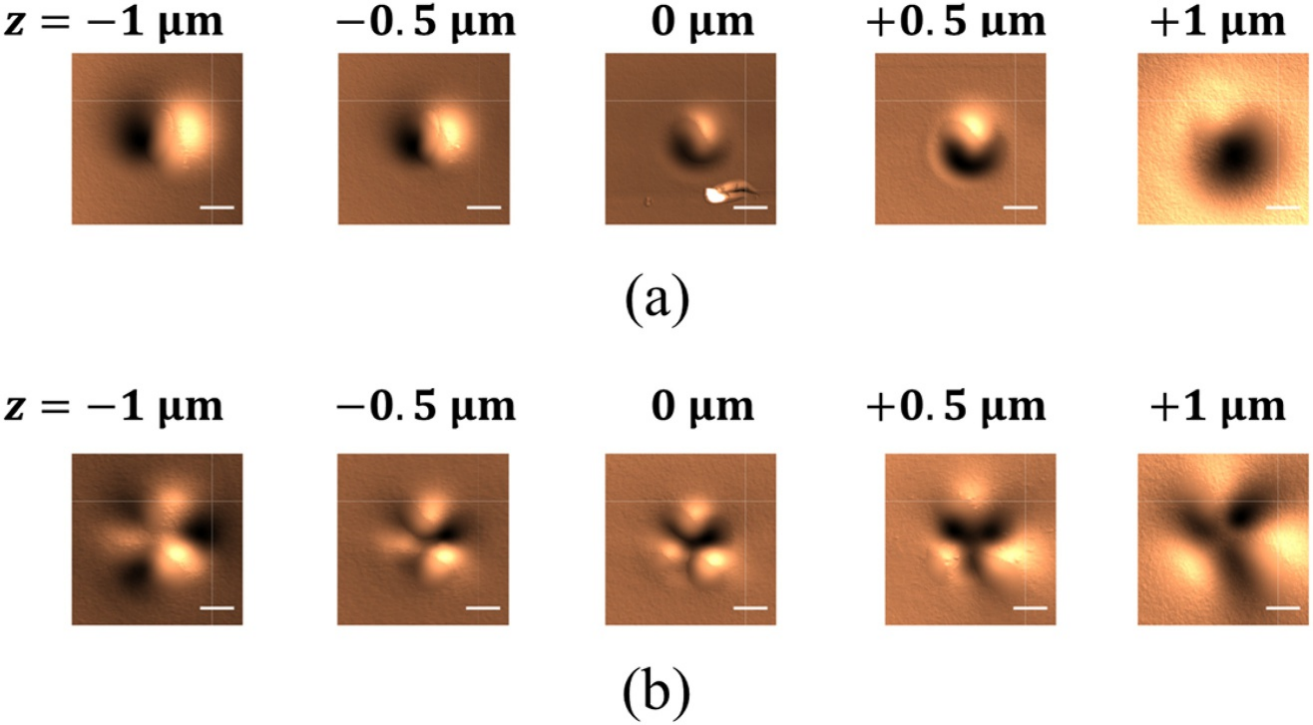
Images showing surface relief structures fabricated at various focusing depths *z* with (a) Bloch and (b) anti‐skyrmions. The solid white line is a 0.5 μm scale bar.

## 3 Discussion

Spatial intensity, polarization profiles, and Stokes parameters of the focused optical skyrmions can be numerically simulated using the vectorial Debye diffraction integral with the fast Fourier transform [39, 40, 41, 42] and conventional finite‐difference time‐domain (FDTD) methods. Note that the power ratio *α* was then fixed to be 1: $\sqrt{2}$.

The longitudinal and transverse electric fields of the left‐handed Bloch skyrmion carry OAMs with *l* = 0 and *l* = 1 associated with SAM‐OAM coupling effects in circularly polarized LG and Gaussian modes. In contrast, the anti‐skyrmion possesses an inverted triangle shaped focused spot arising from the longitudinal electric field with three bright azimuthal lobes and an inverted Y‐shaped dark core, which reflects its star‐shaped polarization texture. Also, its longitudinal and transverse electric fields carry OAMs with *l* = −2 and *l* = −1 (Figure 6). Interestingly, the C‐point defect spot (circular polarization spot represented in *S*_3_) in both Bloch and anti‐skyrmions possesses a subwavelength scale diameter of ∼350 nm (the subwavelength scale C‐point defect spot). Also, Bloch and anti‐skyrmions exhibit a single or triple twisted Möbius‐strip‐shaped electric field around the C‐point defect (Figure 7).

**FIGURE 6:**
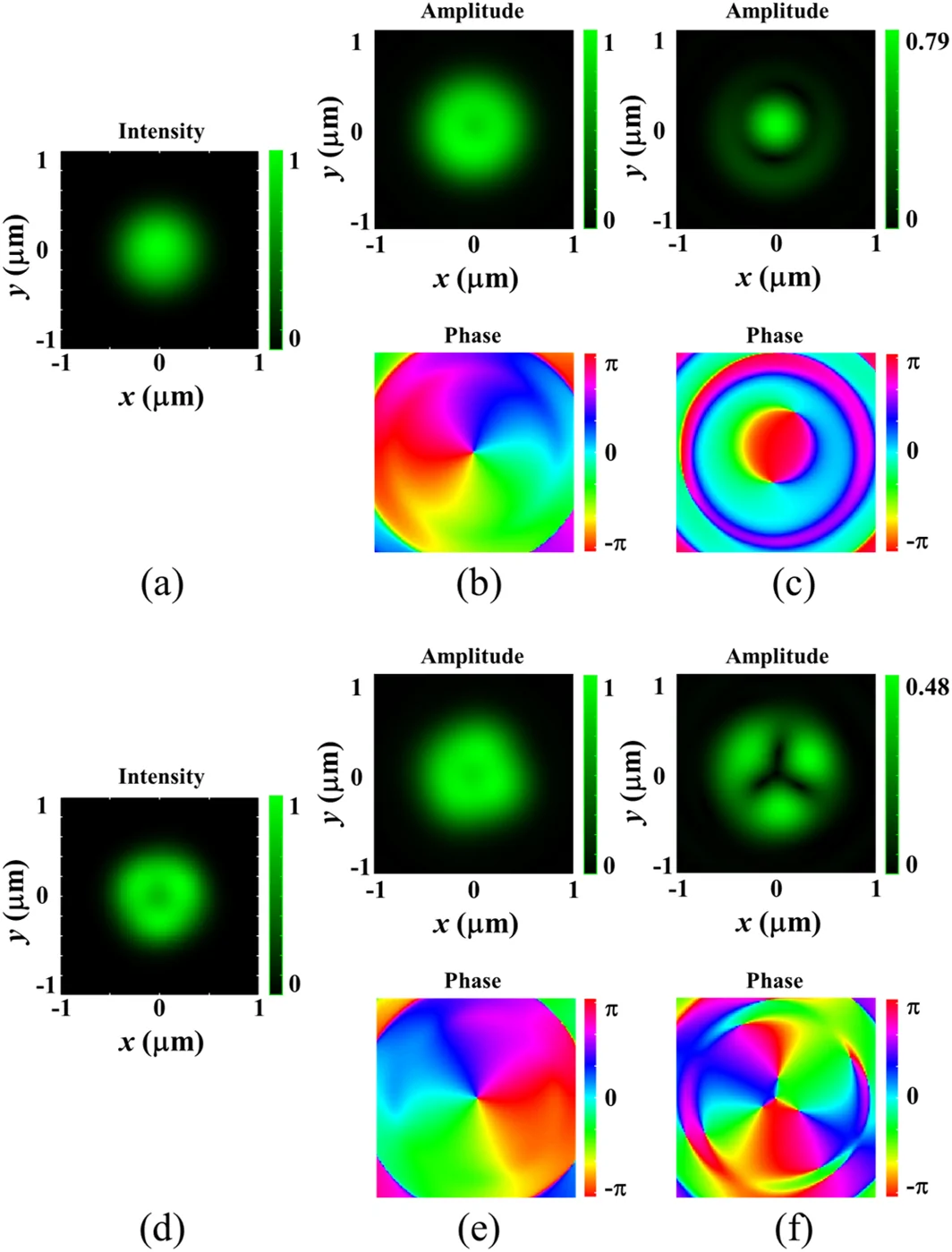
The simulated characteristics of a focused optical skyrmion. (a) Intensity profile, (b) transverse electric field profile, and (c) longitudinal electric field of a Bloch skyrmion. (d) Intensity profile, (e) transverse electric field profile, and (f) longitudinal electric field of an anti‐skyrmion. The power ratio between the Gaussian and LG modes is then fixed at 1: $\sqrt{2}$. The amplitudes of the longitudinal electric fields are normalized by those of the transverse electric fields.

**FIGURE 7:**
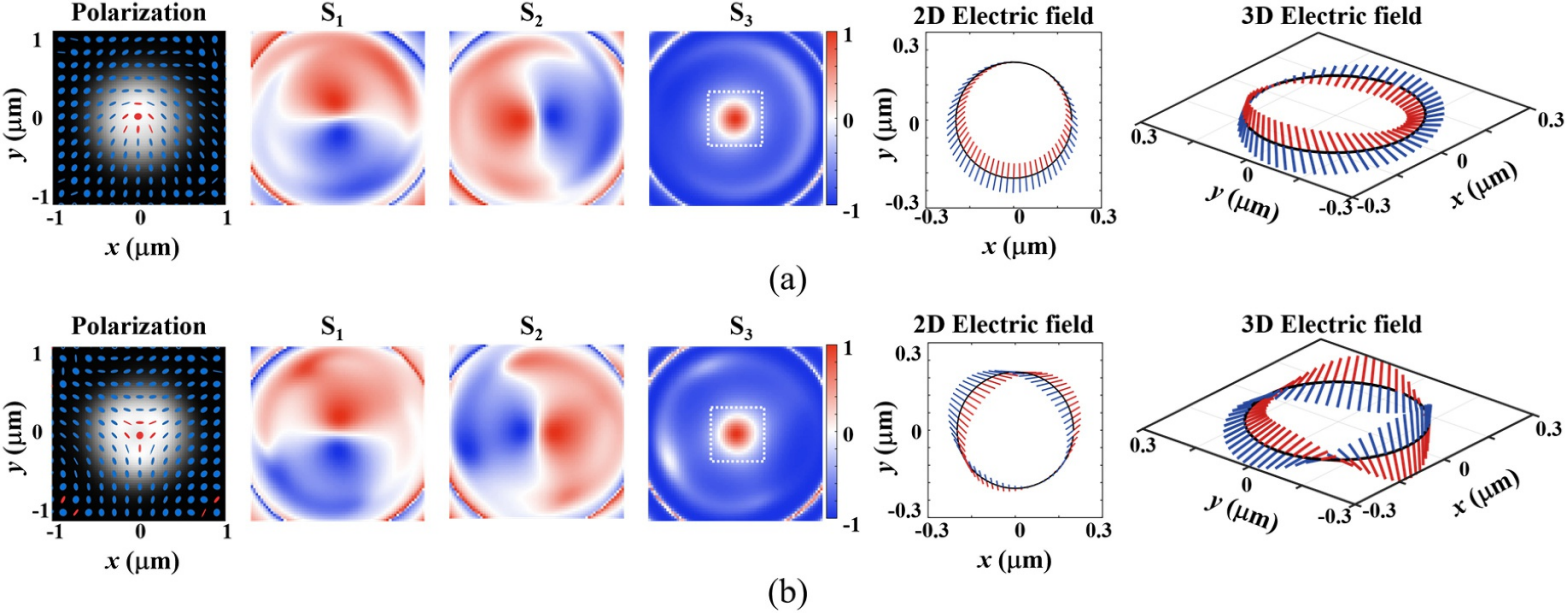
Simulated spatial intensity and polarization profiles, Stokes parameters (S_1_, S_2_, S_3_), and 2D and 3D polarizations (electric fields) (red: positive blue: negative) along the C‐point defect border (white line) in S_3_ (in the white broken square) of focused (a) Bloch and (b) anti‐skyrmions with a power ratio of 1: $\sqrt{2}$ between the Gaussian and LG modes.

The time‐averaged optical scattering force in materials <**F**(*x*, *y*)> is given by,

(3) $$<F\left(x,y,z\right)>\propto \chi_{r}\text{Im}\left(E\times B^{*}\right)+\chi_{i}\text{Re}\left(E\times B^{*}\right)$$

where **E**(*x*, *y*, *z*) (=(*E*_*x*_, *E*_*y*_, *E*_*z*_)) and **B**(*x*, *y*, *z*) (=(*B*_*x*_, *B*_*y*_, *B*_*z*_)) are the electric field and magnetic density flux of the optical skyrmion, respectively, and *χ* (=*χ*_*r*_ + *iχ*_*i*_) is the complex electric susceptibility of azopolymers. It is then assumed that *χ*_*r*_ = *χ*_*i*_ and that the optical skyrmion beam propagates along the *z*‐axis, normal to the material surface. Note that the scattering forces then become significantly stronger as the absorption of azopolymers increases (the increase of *χ*_*i*_); however, the spatial distribution of scattering forces remains qualitatively unchanged regardless of *χ*_*i*_/*χ*_*r*_, as noted in our previous work [43].

The Bloch skyrmion produces clockwise azimuthal forces around the C‐point polarization defect, and it significantly twists azopolymers in a direction toward the higher right edge of the C‐point defect spot (Figure 8b). Furthermore, the scattering force along the *z‐*axis (owing to the longitudinal electric field) pumps up the azopolymers, resulting in the formation of a teardrop‐shaped, clockwise twisted relief with a shape like a Möbius strip. The surface relief reflected significantly the electric field of the Bloch skyrmion around the C‐point defect (Figure 7a).

**FIGURE 8:**
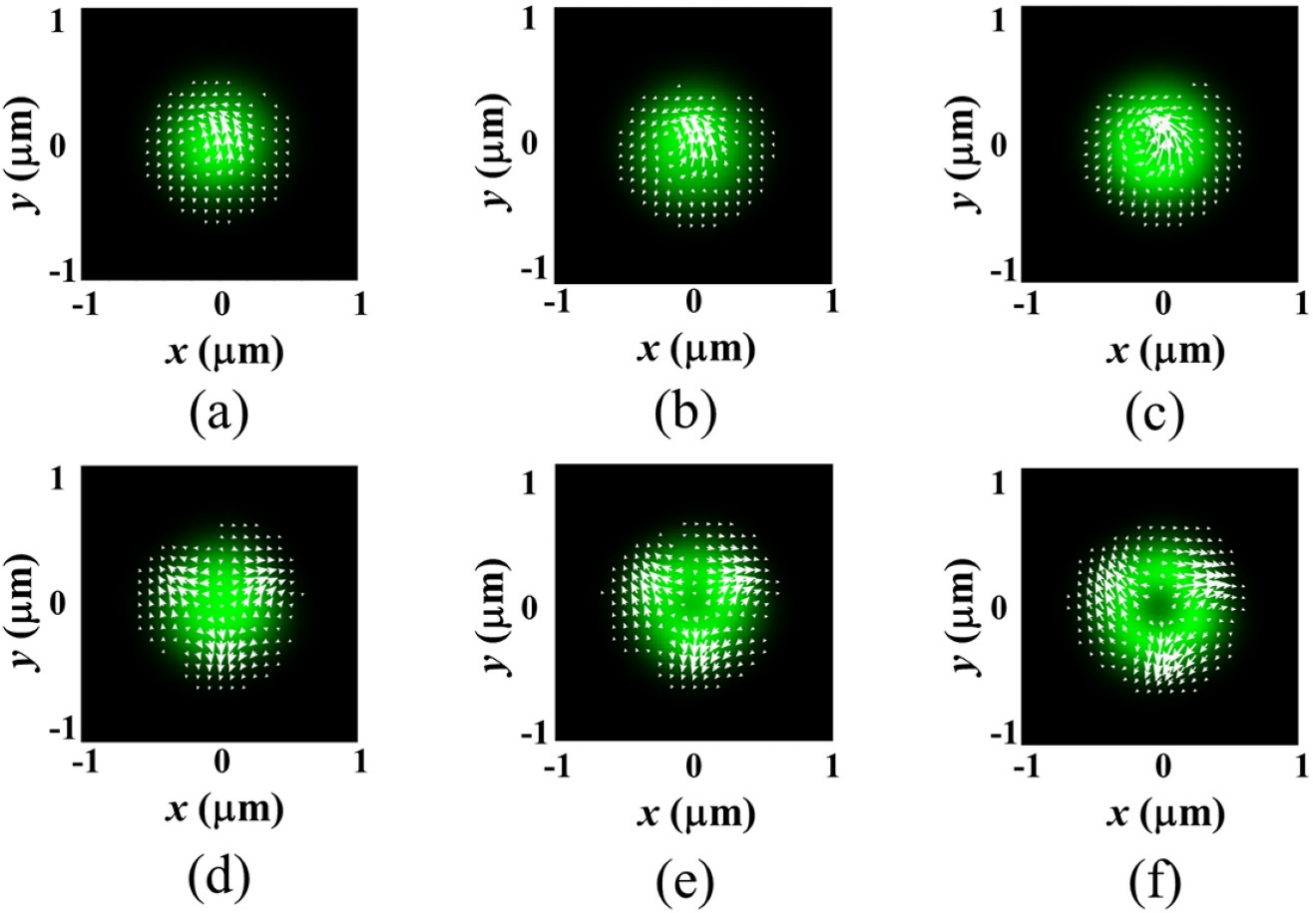
Simulated 2D scattering forces of focused Bloch and anti‐skyrmions in the *x–y* plane. (a) Bloch and (d) anti‐skyrmions with a power ratio of 1:1 between Gaussian and LG modes. (b) Bloch and (e) anti‐skyrmions with a power ratio of 1: $\sqrt{2}$ between Gaussian and LG modes. (c) Bloch and (f) anti‐skyrmions with a power ratio of 1:2 between Gaussian and LG modes.

The anti‐skyrmion produces radial forces (from the longitudinal electric field with three petals) that direct the azopolymer outwards from the C‐point defect so as to form 3 grooves; its azimuthal force outside the C‐point defect is therefore very weak. Thus, the resulting surface relief is achiral (nontwisted), and it resembles the star‐shaped polarization textures (triple twisted electric fields) of anti‐skyrmions (Figures 7b and 8e). It is noteworthy that lowering the value of *α* enables the production of a smaller C‐point polarization defect spot, and this is suggestive of the fabrication of subwavelength scale topological surface relief structures (Figure 8a–f). In this case, the minimum dimensions of the fabricated surface relief will be effectively determined by balancing the diameter and optical intensity of the C‐point polarization defect spot.

These simulations support experimentally demonstrated surface relief structures. 3D scattering forces (represented in Figure 9) highlight that the tightly focused optical skyrmions enable the production of strong longitudinal electric fields, which produce topological (chiral Möbius strip like or achiral three‐folded) surface reliefs in azopolymers with the help of C‐point polarization defects.

**FIGURE 9:**
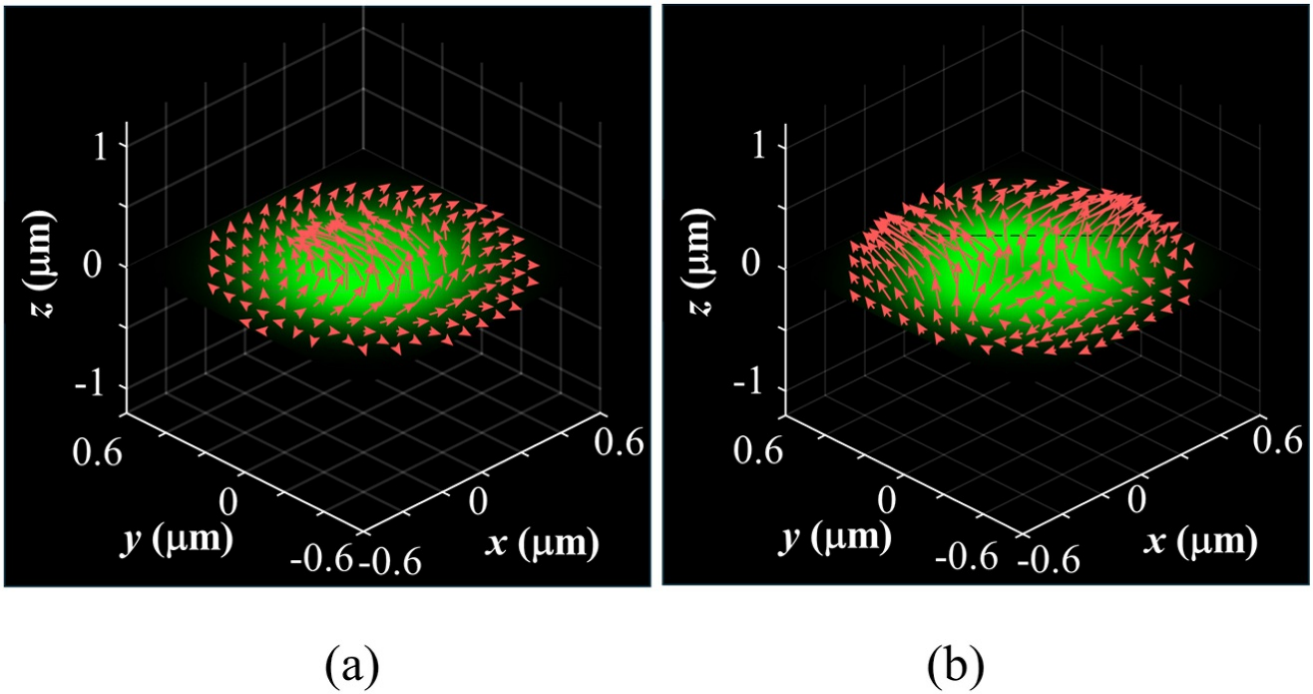
Simulated 3D scattering forces of focused (a) Bloch and (b) anti‐skyrmions in 3D (*x–y–z*) space. The power ratio between the Gaussian and LG modes is 1: $\sqrt{2}$.

Mass transport of azopolymers includes many photochemical processes, such as photoisomerization, molecular reorientation, and viscoelastic transport. In fact, several theoretical models, such as an interaction model between dipoles and the gradient of an optical electric field [44], a photoisomerization force model due to the polymer's elasticity [45], and a diffusion model based on a random walk motion [46], have been proposed. Furthermore, tightly focused OAM fields create a spiral surface relief through azimuthal mass‐transport owing to the interference between the longitudinal and transverse optical fields [38]. We herein discussed mass transport under radiation pressure by assuming the azopolymer to be a passive particle [47]. Even with such a simple analysis, it is possible to gain a qualitative understanding of surface relief formation driven by optical skyrmions.

A diffractive beam‐splitting optical element (DOE) (HOLO/OR diffractive beamsplitter; separation angle: 0.25° × 0.25°) separated spatially a Bloch skyrmion into 9 × 9 skyrmion spots on a focal plane, thus enable the fabrication of multiple surface structures on the azopolymer film in a single exposure. In fact, spatially uniform 9 × 9 reliefs were fabricated with ∼12 μm spacing, and they exhibited the Möbius strip like shape, which reflected topological polarization textures of a Bloch skyrmion (Figure 10).

**FIGURE 10:**
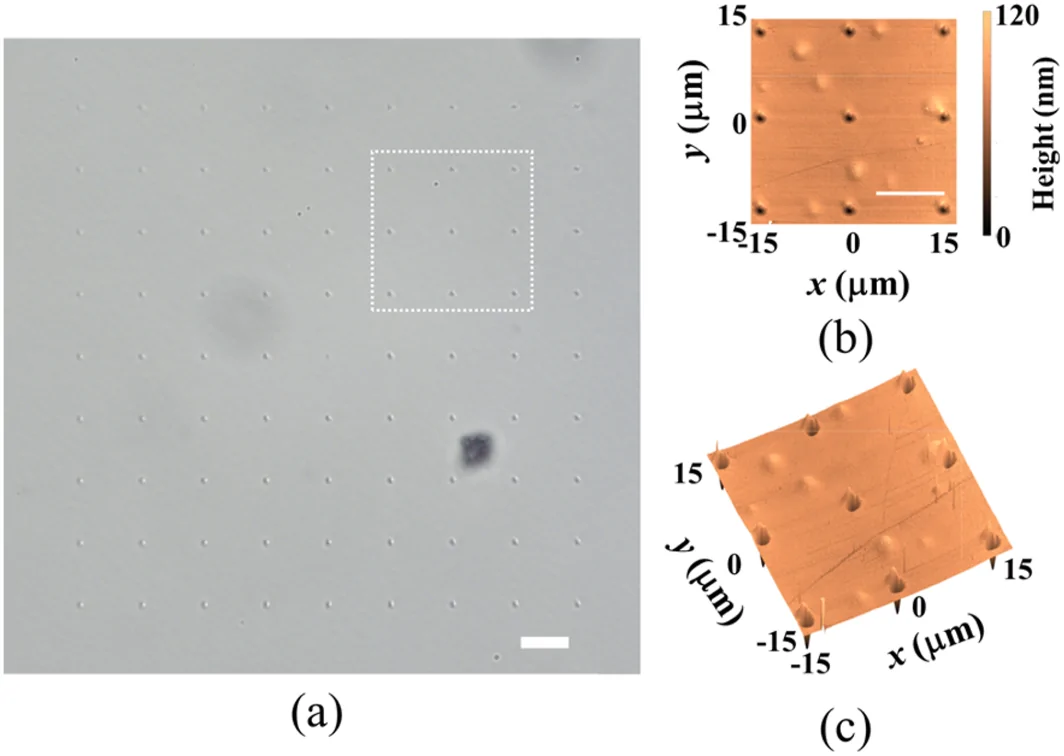
Images showing a 2D surface relief array (9 × 9) fabricated in azopolymers using a beam‐splitting diffractive optical element (DOE) and a single Bloch skyrmion. (a) Optical microscope image. (b) 2D AFM image of a surface relief array (3 × 3) as highlighted by the white box in (a). (c) The corresponding 3D AFM image of the surface relief array (3 × 3). The solid white line is a 10 μm scale bar.

## 4 Conclusion

We have demonstrated the fabrication of topological (chiral Möbius strip like or achiral three‐folded) surface structures on the nano scale beyond the diffraction limit in a photosensitive material (azopolymer film), using tightly focused optical skyrmions. Unlike what was observed under the paraxial condition, the focused optical skyrmions in this work enabled the production of a strong longitudinal electric field around the subwavelength scale C‐point polarization defect. This was due to SAM‐OAM coupling effects. This resulted in the pumping up and twisting of the irradiated material to form surface structures, which fully reflected the topological polarization (electric field) textures of the incident optical skyrmions on the nano scale. This demonstration highlights that optical skyrmions have the capacity for nano scale (subwavelength scale) manipulation of the chirality and topology of materials. This is a process which may be harnessed for chiral nanofabrication and innovative nanoimaging of chiral materials. The fabricated structures shaped like a Möbius strip may also possess further exotic chiral–optical properties, and mechanical properties (such as enhanced wear‐resistance, etc.). Spatiotemporal structured light, such as spatiotemporal optical skyrmions associated with the longitudinal OAM, has been theoretically proposed and experimentally demonstrated [48, 49], and they will further offer additional degrees of freedom for fabricating new chiral or topological structures via nonlinear processes such as multiphoton absorption.

## Author Contributions

**Takashige Omatsu:** conceptualization, methodology, investigation, formal analysis, supervision, resources, project administration, visualization, funding acquisition, writing – original draft, writing – review and editing, data curation, validation. **Soki Hirayama:** methodology, software, data curation, investigation, visualization, writing – review and editing, validation, formal analysis. **Andra Naresh Kumar Reddy:** methodology, validation, data curation, writing – review and editing. **Masahito Okida:** methodology, validation, writing – review and editing, data curation, software. **Atsushi Taguchi:** software, data curation, formal analysis, validation, visualization, writing – review and editing, methodology.

## Funding

This work was supported by the JSPS KAKENHI Grants‐in‐Aid (Nos. JP22H05131, JP22H05137, JP22H05138, JP23H00155, JP23H00270), the JST Core Research for Evolution Science and Technology program (No. JPMJCR1903), and NSTC projetcs (Nos. 115‐2811‐M‐A49‐529, 115‐2112‐M‐A49‐033).

## Disclosure

The authors have nothing to report.

## Data Availability

Data sharing not applicable to this article as no datasets were generated or analyzed during the current study.
